# Progress of Surgery

**Published:** 1903-04-04

**Authors:** 


### Progress of Surgery.

### GENITO-URINARY.

**Varicocele.**—Charles Chassaignac, of New Orleans, has written on this subject.[1] The veins of the pampiniform plexus converge into two or more branches above and these unite at some point above the level of the external abdominal ring to form the spermatic vein. The left vein is generally longer and larger than the right and receives usually two branches from the descending colon. In varicocele some or all of the veins are tortuous, dilated, increased in number, and thicker walled than natural. The thickening is in the middle tunic and apparently the result of slight chronic inflammation. In pronounced cases the testicle is often soft and somewhat shrunken or even atrophied. It is debated whether the atrophy is a result or a coincidence, and whether it is a lack of development or a true shrinkage. The scrotum becomes relaxed, elongated, thinner and less elastic, and often its veins are also varicose. The writer attaches importance to constitutional and even congenital lack of vascular tone as a predisposing cause. Given this predisposition, anything causing greater strain than usual to be thrown upon the veins may act as an exciting cause, *e.g.* constipation, long standing, violent efforts, long-continued sexual excitement, or persistent cough. Masturbation is regarded by most authors rather as a result than a cause. The pressure of a renal tumour may be a cause. Estimates as to the frequency of varicocele vary between 2 and 75 per cent. The estimates depend much upon what degree of enlargement of the veins is considered to be pathological and upon the ages of the persons examined. Only when the veins are enlarged sufficiently to be visible, palpable and the cause of some relaxation of the scrotum, should the condition be considered a diseased one. The author concludes that 10 per cent. is nearly the correct estimate. Bennett claims that about 7 per cent. of men have varicocele. Probably the right side only is involved in about 5 per cent. of all cases. Varicocele has been seen in boys under 10 years of age, but over half the cases are found at from 15 to 25 years of age. The effects may be classified as mechanical, reflex, or mental. The mechanical are a sensation of weight and dragging pain and tenderness in the testicle, cutaneous irritation and encumbrance owing to the size of the swelling. The reflex effects comprise neuralgias in the testis only or radiating to the penis, back, perineum, thighs, etc. The former may be very intense and lead patients to ask for castration. Disproportionate anxiety is characteristic of the disorder. The disease is apt to increase gradually to middle life, but after that gives much less trouble. Radical treatment promises a positive removal of the abnormality. Shrunken testicles have been known to grow and resume a natural state after the operation. Sometimes, however, neuralgic pains persist, and impotence if present, cannot with certainty be relieved. The author describes three methods of operating only, viz., by subcutaneous ligature, by excision through a scrotal incision, and by excision in the groin. He deems a sacrifice of scrotal tissue to be either insufficient or superfluous. If the scrotum is much elongated the longitudinal incision may be sutured in a transverse direction. The conclusions of Bennett with regard to the division of the spermatic artery are quoted, but the preservation of the artery is rather advocated. Bennett says that only the complete division of the whole spermatic cord, with the exception of the vas deferens, gives certainty of complete cure. Chassaignac prefers the scrotal incision.

**Imperfectly-descended Testis.** — W. McAdam Eccles[2] has discussed the question as to the value of the imperfectly-descended testis. The condition is often associated with a hernia into the unclosed processus vaginalis requiring operation, and proper treatment of the testis in the operation depends upon the value of the organ. The imperfectly-descended testis does not atrophy, but as a rule it develops imperfectly. One testicle normally developed and descended is probably capable of exerting all the influence necessary for perfect development bodily, mentally, and sexually. If, however, both testes have descended and developed imperfectly there will almost certainly be deficient development of the individual, though this is not always the case. If one testis only has properly descended it does not as a rule hypertrophy, as if it were more functionally active than normal. The question arises, does the imperfectly-descended organ in such cases help in any way in developing the individual along normal lines, even though it remains incapable of producing spermatozoa. In normal testes, about puberty, cells, termed interstitial cells, develop in the spaces between the seminiferous tubules. In retained testes these cells seem to be peculiarly abundant even long before 15 years of age. These cells may have the duty of providing an internal secretion which is required for the proper growth of the body. If this is the case it is obvious that the imperfectly-descended testes is an organ worth preserving. When such a testis is exposed in an operation for hernia there are at least three ways of dealing with it to consider, viz., by transplanting it into the scrotum, by returning it into the abdomen, or by removing it. Unfortunately there is a singular lack of evidence that testicles which have been transplanted into the scrotum ever grow and develop properly, and this points strongly to the conclusion that such growth very rarely occurs. Also many of such transplanted testes do not remain in the scrotum long but become retracted to a level not much below that at which they were originally situated. Therefore there seems to be but little gained by transplanting testes the development of which has been arrested. The plan of returning the testis into the abdomen has much to recommend it. There is no evidence that the organ, if so returned, is more liable to develop malignant disease than a normally placed testicle. Removal of the organ is the most satisfactory plan from many points of view, though against the plan is the possibility that the internal secretion of the gland is of value in the economy. Eccles therefore only recommends transplanting the testicle if it can be done perfectly easily; if not he considers it preferable to return the organ into the abdomen or to remove it. If both testes are arrested it will not be wise to remove either lest too little internal secretion be then produced. In such a case the testicle on the hernia side should be replaced in the abdomen.

**Tuberculosis of the Testis, Prostate, and Seminal Vesicles.**—Sir Thomas Myles opened the discussion on this subject at the Manchester meeting of the British Medical Association.[3] He stated that he was convinced that tubercle of the prostate and vesiculæ seminales was almost invariably secondary to tubercle of the testis, and that tubercle of the bladder was often secondary to tubercle of the kidney and rarely primary. On the other hand tuberculosis of the testis was often a primary disease and occurred where no trace of tubercle could be detected in the lungs or elsewhere. It was contended that for such a primary nodule of tubercle in the testis complete orchectomy was not justifiable. In cases in which the total operation only seemed worth considering most probably general infection had already taken place. For isolated nodules of tubercle in the epididymis the author's practice was to resect the mass *in toto* without any regard to anatomical relations, swab the wound out with a ten per cent. formaline solution and then close it. If that failed he advised total extirpation. If there was disease of the prostate and vesicles present also he did not place much reliance on operations. In those cases in which the testicle was much enlarged and suppurating, he removed it, even if there was also disease of the prostate, in order to place the patient in a better condition for resisting general infection. In cases involving both testes, sometimes total castration would have to be recommended. In cases showing lung or other infection operation was inadmissible. In tubercular disease of the bladder he considered the outlook from operative interference was not encouraging and he hesitated to recommend operation, but in some cases would operate and subsequently drain the bladder through the perineum. Horwitz[4] has contributed a paper on tubercular disease of the testicle based upon an analysis of 96 operations for the relief of the disease. He points out that heredity is a rare cause, but is undoubted, for the disease has been observed in infants a few days old. Slight injuries are more likely to cause the disease than severe ones, and gonorrhœa favours it even without giving rise to epididymitis. The disposition of the spermatic artery favours the deposit of tubercle bacilli in the epididymis. It is not rare for the testicle and epididymis to be affected in general tuberculosis. The disease usually has a very slow onset, sometimes beginning in the epididymis, sometimes in the testicle, and the degree of implication of the vas deferens varies much, sometimes being involved throughout, at other times in part. The diagnosis often presents difficulties and sometimes with advantage tuberculin may be used as a diagnostic aid, or hydrocele fluid may be injected into guinea-pigs. The writer states that the removal of the vas deferens, which is best performed through an incision above Poupart's ligament, has often led to complete subsidence of the disease in the vesiculæ, bladder, and prostate. The removal of the vesiculæ is a very severe operation and should not be performed unless it is certain that they are extensively diseased. To establish such a diagnosis a long urethroscopic tube should be passed into the prostatic urethra and the prostate massaged so that the contents of the vesicles are received directly into the tube. Failing that the bladder contents should be examined after the massage. Only if the bacilli are found in one of these ways to come from the vesicles should they be removed. The smegma bacillus must be prevented from contaminating the collected specimen by using a catheter if the urinary deposit has to be examined.

[1] Med. Rec., Oct. 18, 1902, p. 603. [2] Brit. Med. Jour., Oct. 25, 1902, p. 1314. [3] Ibid., p. 1307. [4] Vide Abst. Centralb. f. Chir., Sept. 6, 1902, p. 945.

*(To be continued.)*

